# Top–Down Proteomics of Human Saliva, Analyzed with Logistic Regression and Machine Learning Methods, Reveal Molecular Signatures of Ovarian Cancer

**DOI:** 10.3390/ijms242115716

**Published:** 2023-10-28

**Authors:** Francesca Scebba, Stefano Salvadori, Silvia Cateni, Paola Mantellini, Francesca Carozzi, Simonetta Bisanzi, Cristina Sani, Marzia Robotti, Ivana Barravecchia, Francesca Martella, Valentina Colla, Debora Angeloni

**Affiliations:** 1 Health Science Interdisciplinary Center, Scuola Superiore Sant’Anna, Via G. Moruzzi, 1, 56124 Pisa, Italy; f.scebba@santannapisa.it; 2 Institute of Clinical Physiology, National Research Council, Via G. Moruzzi, 1, 56124 Pisa, Italy; stefsa@ifc.cnr.it; 3 Center for Information and Communication Technologies for Complex Industrial Systems and Processes (ICT-COISP), Telecommunications, Computer Engineering, and Photonics Institute (TeCIP), Scuola Superiore Sant’Anna, Via G. Moruzzi, 1, 56124 Pisa, Italy; s.cateni@santannapisa.it (S.C.); v.colla@santannapisa.it (V.C.); 4 Istituto per lo Studio, la Prevenzione e la Rete Oncologica (ISPRO), Via Cosimo il Vecchio, 2, 50139 Firenze, Italy; p.mantellini@ispro.toscana.it (P.M.); f.carozzi@ispro.toscana.it (F.C.); s.bisanzi@ispro.toscana.it (S.B.); c.sani@ispro.toscana.it (C.S.); 5 Ph.D. School in Translational Medicine, Scuola Superiore Sant’Anna, Via G. Moruzzi, 1, 56124 Pisa, Italy; m.robotti@santannapisa.it; 6 The Institute of Biorobotics, Scuola Superiore Sant’Anna, Via G. Moruzzi, 1, 56124 Pisa, Italy; barravecchia.ivana@gmail.com; 7 Breast Unit and SOC Oncologia Medica Firenze—Dipartimento Oncologico, Azienda Usl Toscana Centro, Ospedale Santa Maria Annunziata, Via dell’Antella, 58, 50012 Firenze, Italy; francesca.martella@uslcentro.toscana.it

**Keywords:** biomarkers, breast cancer, logistic regression, machine learning, mass spectrometry, ovarian cancer, proteomics, saliva, SELDI-TOF-MS

## Abstract

Ovarian cancer (OC) is the most lethal of all gynecological cancers. Due to vague symptoms, OC is mostly detected at advanced stages, with a 5-year survival rate (SR) of only 30%; diagnosis at stage I increases the 5-year SR to 90%, suggesting that early diagnosis is essential to cure OC. Currently, the clinical need for an early, reliable diagnostic test for OC screening remains unmet; indeed, screening is not even recommended for healthy women with no familial history of OC for fear of post-screening adverse events. Salivary diagnostics is considered a major resource for diagnostics of the future. In this work, we searched for OC biomarkers (BMs) by comparing saliva samples of patients with various stages of OC, breast cancer (BC) patients, and healthy subjects using an unbiased, high-throughput proteomics approach. We analyzed the results using both logistic regression (LR) and machine learning (ML) for pattern analysis and variable selection to highlight molecular signatures for OC and BC diagnosis and possibly re-classification. Here, we show that saliva is an informative test fluid for an unbiased proteomic search of candidate BMs for identifying OC patients. Although we were not able to fully exploit the potential of ML methods due to the small sample size of our study, LR and ML provided patterns of candidate BMs that are now available for further validation analysis in the relevant population and for biochemical identification.

## 1. Introduction

Worldwide, ovarian cancer (OC) is the most lethal malignancy of the female reproductive tract [1]. There is inconsistency in the availability of and access to treatment for OC. Additionally, outcomes are complicated by poor understanding of the disease, which is characterized by complex epidemiology, histopathology, and genetic features [2]. OC is a heterogeneous disease comprising several types and subtypes [3,4], and the extra-ovarian origins of epithelial OC contribute to its intricacies.

Despite increasingly radical surgical approaches and huge efforts put into new, targeted therapeutic agents, the prognosis for patients with OC has hardly improved in the past three decades, and two-thirds of women still die within 10 years of diagnosis. This is mostly due to late diagnosis: nearly 70% of patients are diagnosed with advanced disease (stage III or IV) and have a five-year survival below 20% [5]. In contrast, the five-year survival of patients diagnosed at stage I exceeds 90%. A yearly screening test that could detect tumors below 0.5 cm in diameter would reduce mortality by 50% [6]. Therefore, strategies for curing OC require identifying new biomarkers (BMs) to achieve two main goals: to accurately detect OC early, at a point when outcomes could be improved, and to perform a better stratification of patients with full-blown disease.

On the front of early detection, at least three high-quality studies evaluated the effect of annual screening in asymptomatic women not known to be at high risk for OC [7,8,9]. None found that screening significantly reduced OC mortality. In 2018, the US Preventive Services Task Force (USPSTF) updated its guidelines to recommend against screening for OC in “asymptomatic women with no family history of cancer” [10] to avoid potential serious harm from false positive results (e.g., unnecessary surgical removal of the ovary).

Presently, screening procedures include measuring the serum cancer antigen 125 (CA125), which is one of the most common serum BMs used in the diagnosis of OC. However, CA125 is not specific to OC, as its level also increases in adenomyosis, uterine myoma, endometrial pathology, and endometriosis of the ovary [11]. Moreover, CA125 increases in about 80% of all OC and in 50% of stage I epithelial OCs [12,13]; therefore, using CA125 as the only diagnostic BM will miss those that do not express this antigen. However, serum BMs other than CA125 are not currently used for screening in clinical practice due to their low sensitivity or specificity [14]. 

At the same time, the current literature agrees that multi-BM panels perform better than single markers for more personalized treatment in the context of precision medicine [15,16] and for the detection of OC [2,17]. However, attention must be paid to the type of markers combined for diagnostic purposes in relation to the intended use of the panel. For example, it was shown that markers discovered in diagnostic samples are significantly differentially expressed only when the tumor becomes large or clinically apparent; therefore, such markers may have little value for early detection [18,19].

Thus, the need for more sensitive and specific tests that will minimize false positives, predict metastasis, and provide better clinical management of OC patients remains unmet [16].

Among body fluids, saliva is a relatively simple, accurate, safe, and economical material that can be tested for clinically significant molecules [20]. Saliva released by the major salivary glands consists of 99% water containing inorganic and organic species, including secretion and putrefaction products, lipids, over 2400 proteins, metabolites, components of the microbiome and abundant, stable extracellular coding and non-coding RNA species [21]. It can be collected without medical intervention, properly stabilized at a minimal cost, and stored and shipped from the collection site to the testing site. Some of the molecule types characterized in saliva are candidate BMs for cancer diagnosis, prognosis, drug monitoring and pharmacogenetic studies, and a few such candidates have been validated in multicenter studies with large sample sizes and standardized protocols [22].

Until now, only one study has reported the analysis of saliva proteome for detection of OC BMs [23,24]; however, this study only compared OC patients to healthy women. Therefore, we set out to (A) search for candidate biomarkers of OC using a broad, unbiased approach based on high-throughput proteomics technologies, using saliva as a test fluid from three cohorts of women: (1) patients with OC at various stages, (2) breast cancer (BC) patients and (3) healthy subjects (HS); and (B) analyze the results using both a traditional statistical approach based on logistic regression (LR) and a machine learning (ML) approach for pattern analysis and variable selection (VS) [25,26]. Our purpose was to highlight relevant combinations of candidate BMs (signatures) for the diagnosis of OC and possible re-classification of disease. HS and BC served as reference groups. BC patients were introduced to rule out molecular species possibly shared by cancer patients in view of similarities among the diseases both in terms of gene expression and genetic origins [27,28,29].

We opted for a two-fold approach to data analysis because with respect to standard techniques, ML-based approaches also consider highly non-linear correlations between the potential input variables and the classification task and, if properly tuned, allow the selection of variables conveying the greatest amount of information by reducing the sensitivity to the particular dataset [30]. For the classification tasks, different approaches compatible with the small amount of available data were explored.

In this work, we show as a proof of principle that saliva is an informative test fluid for an unbiased proteomic search of candidate BMs that can discriminate between OC patients and other cohorts. LR and ML analysis provided patterns of candidate BMs that are now available for further validation analysis in the relevant population and for biochemical identification. The small sample size prevented the full exploitation of ML data analysis; however, the results obtained are robust, suggesting that increasing the number of patients will improve the performance.

## 2. Results

### 2.1. Proteome Profiling of Saliva Samples

**Figure 1:**
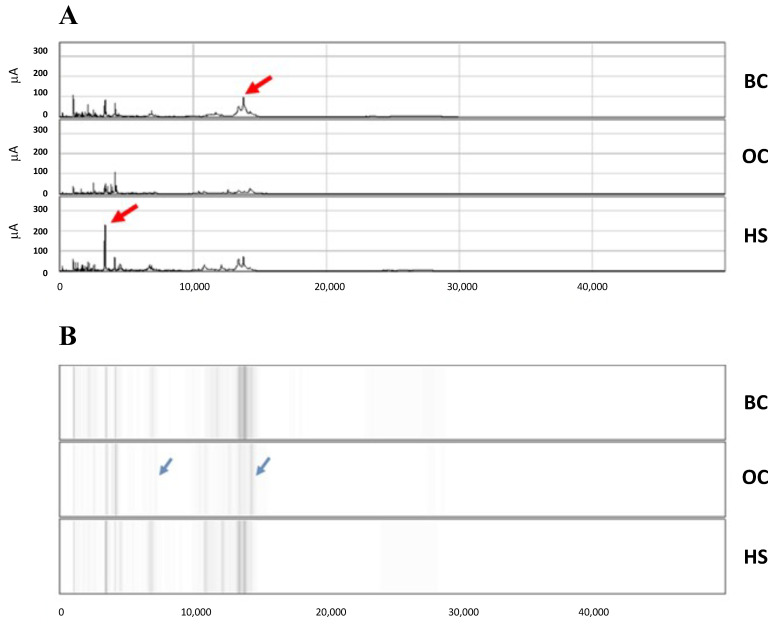
Typical results with mass spectra in the range 2–50 kDa are shown for each cohort of subjects, OC and BC patients, HS. Differences were mostly quantitative (red arrows) but also qualitative (blue arrows), especially in the spectra of OC samples. (**A**) Chromatograms. (**B**) Virtual gels.

Saliva samples of women belonging to three cohorts (OC; BC; HS), were examined with Surface-Enhanced Laser Desorption Ionization-Time of Flight-Mass Spectrometry (SELDI-TOF-MS) using CM10 (cationic exchange surface) ProteinChips to reveal qualitative and quantitative differences in the ionic proteome profile. Typical results with mass spectra in the range 2–50 kDa are shown for each group in the form of chromatograms (Figure 1A) and virtual gels (Figure 1B). Differences between samples were mostly quantitative, although some, especially in the spectra of OC samples, were also qualitative (Figure 1, arrows).

### 2.2. Ionic Species Differentially Expressed in the Training Set

To search for differentially expressed peaks, spectra acquired from a training set of 147 samples were analyzed with ProteinChip Data Manager 3.5 software (BioRad Laboratories, Segrate (MI), Italy), using *m*/*z* values and their relative intensities as variables in the range 2–50 kDa. The software produced a cluster list of 77 peaks that were common to at least 80% of all saliva samples of the training set (Table 1).

Statistical analyses performed with a non-parametric Mann–Whitney *U* test for the comparison between two groups, respectively, showed that among the 77 ionic peaks of the cluster list, 33 were differentially expressed (Table 2).

Next, to identify candidate biomarkers specific to OC, we compared the three cohorts of subjects to one another. We included BC samples in the study to rule out possible biomarkers shared by the two gynecological cancers and possible non-specific biomarkers associated with cancer, such as inflammation markers. 

Peaks 9, 28, 29, 48 and 57 discriminate OC both from BC patients and from HS but do not discriminate BC from HS. Of those, peaks 9, 28, and 29 are over-expressed in OC, while peaks 48 and 57 are under-expressed in OC compared to BC and HS.

Peaks 4, 5, 8, 10, 22, 24, 26, 27, 49, 67, 68, and 69 are shared by OC and BC, with expression levels similar in the two groups.

Peak 22 distinguishes BC against HS.

Ionic species around 13,000 (peaks 58 to 61) were under-expressed both in OC and BC compared to HS of the training set; in particular, peak 60 was significantly over-expressed in HS against all cancer groups, altogether suggesting the reduction or loss of tumor-suppressor species in both forms of cancer. They were not further analyzed in the testing set.

### 2.3. Ionic Species Differentially Expressed in the Testing Set

To validate the results obtained with the training set, we analyzed with CM10 an independent set of samples belonging to the cohorts of HS, BC and OC patients.

Table 3 shows the 18 significantly differentially expressed peaks obtained with the testing set.

Six of the peaks highlighted from the training set, namely peaks 20, 34, 48, 58, 59 and 60, were confirmed here both for significance and trend of variation (Table 3). In particular, peak 48 was confirmed to be over-expressed in OC compared to BC.

**Figure 2:**
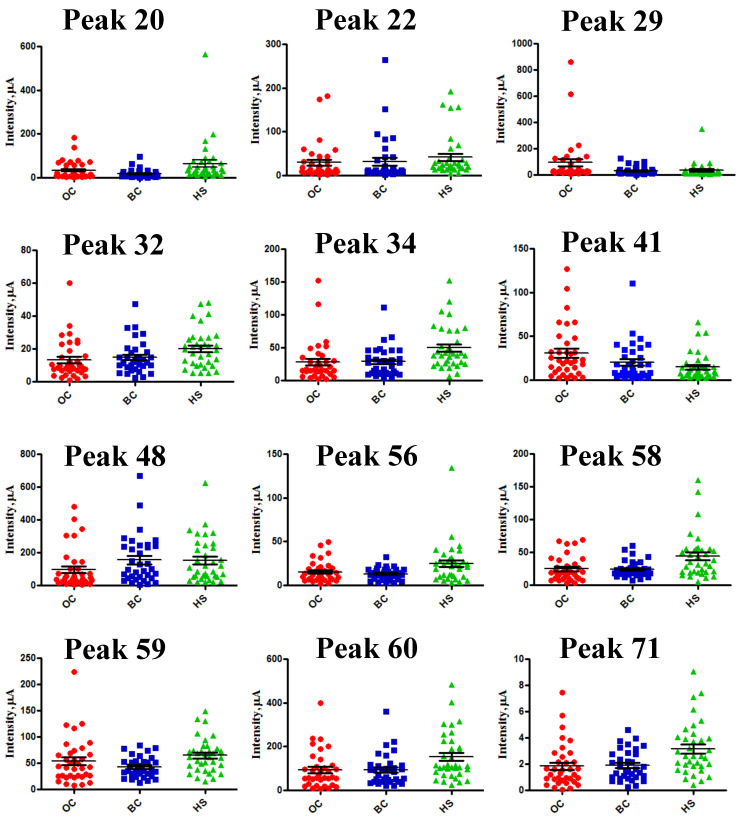
Ionic species differentially expressed in the testing set. Scatter plots of intensity comparison among *m*/*z* peaks of the three sample cohorts for the 12 peaks included in the LR analysis. Red: OC cohort; Blue: BC cohort; Green: HS cohort. Cardinal numbers of peaks refer to *m*/*z* species listed in Table 1.

Six more peaks (peaks 22, 29, 32, 41, 56 and 71) showed the same trend as above but lost statistical significance while still remaining close to the threshold, which was likely because of a smaller number of samples in the testing set (Table 3 and Figure 2). All 12 peaks were included in the logistic regression (LR) analysis performed to identify the best-fitting OC biomarker signature. Scatter plots of the comparisons are shown in Figure 2.

### 2.4. Logistic Regression (LR)

We then applied an LR analysis by “stepwise backward” selection on the training set, using the 12 peaks achieved as above, to obtain receiver operating characteristic (ROC) curves able to measure the accuracy of the diagnostic test in terms of sensitivity and specificity.

**Figure 3:**
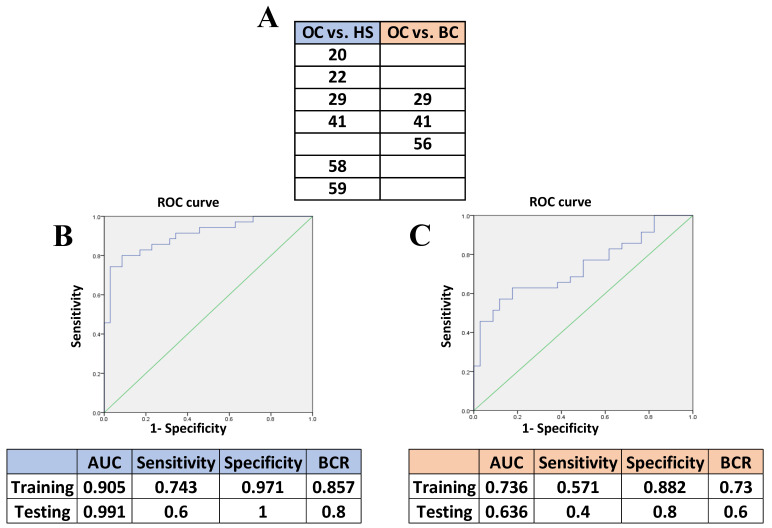
Results of LR analysis to discriminate OC from BC patients and HS. (**A**) List of the discriminating protein peaks defined by LR for each comparison. The results show that saliva contains ionic species able to discriminate OC patients. Cardinal numbers of peaks refer to *m*/*z* species listed in Table 1. Peaks listed here and not present in Table 3 (which lists only statistically significant peaks from the validation step) were among those whose trend was confirmed in the testing set but lacked statistical power; however, they were used to build the LR model. (**B**) ROC curve for the comparison OC versus HS, with relative area under the curve (AUC), sensitivity and specificity. (**C**) ROC curve for the comparison of OC versus BC patients, with relative AUC, sensitivity and specificity. BCR: Balanced Classification Rate. Green line: random classifier curve. Blue line: actual test curve.

This analysis generated a panel of six peaks with the highest accuracy in discriminating OC from HS (Figure 3A). The ROC curve relative to the comparison between OC and HS showed 0.971 specificity and 0.743 sensitivity with 0.905 AUC. The best cut-off value, measured with the Youden index, was 0.714. The predictive model created with the training set was then used to validate the biomarker panel with the testing set. Comparing OC vs. HS, we obtained 60% sensitivity and 100% specificity, suggesting that all HS were correctly classified (true negative); the AUC was 0.991 (Figure 3B).

The same type of analysis to discriminate OC from BC with the highest accuracy generated a further panel of three peaks (Figure 3A). The ROC curve relative to the comparison between OC and BC showed 0.882 specificity and 0.571 sensitivity; the AUC was 0.736 (Figure 3C). When the predictive model was used on the testing set, we obtained 0.8 specificity and 0.4 sensitivity with AUC of 0.636 (Figure 3C).

### 2.5. Machine Learning (ML)

In addition to the traditional approach of LR, we investigated whether ML might provide additional insights. The proposed approach (see Methods) was tested on a dataset including 77 input variables (Table 1).

To evaluate the performances of both VS algorithms and classifiers, we considered the entire initial dataset. A total of 100 classification simulations were performed for each subset by periodically mixing the order of patients analyzed, thereby varying the composition of the training and testing sets. The dataset was shuffled and then divided into a training set and testing set. The training test included 75% of the whole dataset, while the testing set included the remaining 25%.

These tests were performed with four classifiers, considering the different sets of input selected variables. The results, in terms of mean Balanced Classification Rate (BCR), mean sensitivity, mean specificity and mean AUC are shown in Table 4, Table 5 and Table 6.

Moreover, Table 7 shows the classifiers applied to the initial dataset, including all available variables without any selection.

Finally, Table 8 shows a summary of the results and the percentage gain in terms of performance obtained using the VS algorithm rather than by exploiting all available variables.

## 3. Discussion

This work met its main goals of identifying candidate BMs differentially expressed in OC patients through an unbiased, high-throughput proteomic approach and using saliva as a suitable test fluid while comparing OC patients, BC patients and HSs.

To our knowledge, this is the first work to identify salivary proteomic signatures of OC and BC concomitantly.

We used an unbiased approach consisting of recruiting all OC patients that approached our reference clinic regardless of the different stages of the disease (see Section 4). MS in the molecular range 2–50 kDa was applied for a high-throughput proteomic study, and LR was used to identify the panel of candidate BM with the best score in terms of specificity and sensitivity.

**Figure 4:**
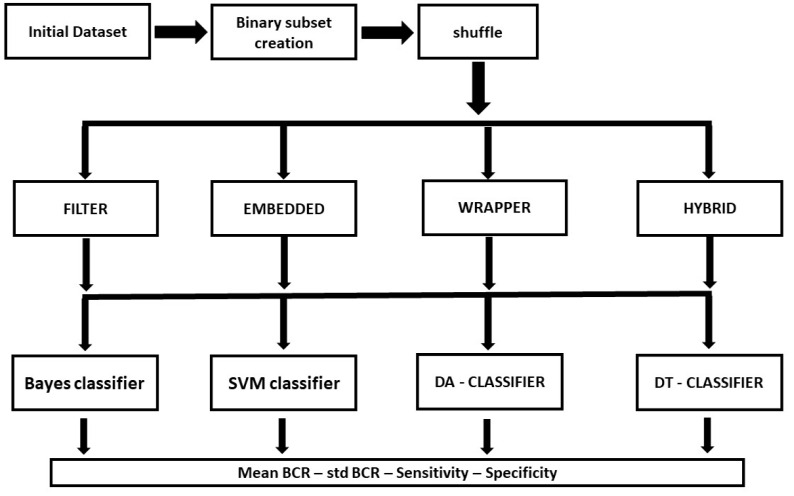
Schematic representation of the proposed logical procedure for ML analysis.

The comparative proteomic analysis showed several ionic species to be differentially expressed in a statistically significant way among the three cohorts of subjects analyzed, first in the training set (Table 2) and then in the testing set (Table 3). Looking for a signature that could effectively discriminate OC patients from HS, LR showed that six ionic species (peaks 20, 22, 29, 41, 58, 59) provided the best combination (Figure 4), and the predictive model created with the training set and validated with the testing set obtained 60% sensitivity and 100% specificity, suggesting that all HS were correctly classified as true negative.

The specificity obtained with the testing set is particularly meaningful considering that a potential harmful consequence of OC screening is unnecessary, invasive diagnostic procedures in cases of false positives.

In addition, since we included BC patients in the study to rule out possible markers common to the two types of gynecological cancers, this work generated a pattern of three candidate biomarkers (peaks 29, 41, 56) that discriminate OC from BC patients (Figure 4).

Moreover, it is interesting to note that even though the comparison between OC vs. BC yielded 40% sensitivity and 80% specificity, peaks 29 and 41 still specifically characterized OC patients vs. both BC and HS (Figure 4A), suggesting that these markers might provide tools for the accurate diagnosis of OC patients against BC.

Furthermore, we extended our analysis to ML, which is an approach that is increasingly taking off for reprocessing data derived from biomedical research and other fields as an alternative to traditional statistical approaches [31,32,33]. In fact, statistical methods are usually top–down approaches in which the relationship between input and output is assumed to be known by the user, who creates a mathematical model. In contrast, ML methods are bottom–up approaches. No assumptions are made about the model that links inputs and outputs, and the algorithm develops a model whose main goal is prediction. The resulting models are often complex, and some parameters cannot be directly estimated from the data. Compared to other methods, ML algorithms can handle a larger number of variables but also require a larger sample size for analysis. ML is able to highlight the complex interactions between all variables while also eliminating those with minimal contribution to outcome prediction.

With the above-mentioned methodology, we demonstrated (Table 8) the effectiveness of performing VS as a starting analysis of our data: BCR values are lower when entering the entire dataset with all 77 variables included (shuffled at each iteration for 100 simulations) instead of using only the most suitable ones.

Among all the VS algorithms used, the best performing approach was the embedded one (Table 4, Table 5 and Table 6); further, since the VS algorithm is embedded in the Decision Tree (DT), the best classifier consequently was the DT. The use of a DT-based classifier has the additional advantage of being a “white box” model, which is easy to interpret, as it is a chain of simple if–then rules. Each node of the DT is connected to an input variable, a branch is related to a range of values, and finally, leaf nodes are associated with both classes.

Our binary comparisons and results show that only variable 3 can discriminate OC patients from BC patients and HS (Table 8). In contrast, variables 20 and 34 are less specific to the OC group because they distinguish the two groups of cancer patients (OC + BC) from HS but not from each other. Although identifying variables to specifically distinguish BC patients from HS was not our main purpose, it is interesting to point out that the remaining variables in Table 8 (i.e., peaks 19, 21, 22, 49, and 54) do distinguish BC from HS.

The variables obtained by VS have the power of classification and are considered an impactful result, as they allowed us to reduce the dataset complexity and consequently to focus only on those variables that better discriminate between the groups of subjects. Furthermore, the accuracy index was high enough, considering the small number of patients available for this study: a larger population would make the training phase easier and more efficient for applying the results to different datasets.

In this study, when considering Subset 2 (OC vs. HS), the two approaches to data analysis, that is LR and ML, yielded results that are not completely overlapping in terms of the identity of variables selected to compose the OC signature, of their number in the signature and of their sensitivity and specificity (see Figure 3 and Table 5). This was expected based on an ever-increasing body of literature dedicated to comparing the modeling performance of the two approaches, which both have advantages and disadvantages.

As already mentioned, an important advantage of ML over conventional statistical methods (like LR used here) is that the various ML algorithms do not require data to conform to statistical assumptions, such as the independence of observations and the avoidance of multicollinearity of independent variables [34]. Another often-cited advantage of ML is that it can model complex, non-linear relationships between the predictors and the outcome [35,36], while the optimal application of the LR model provides better sensitivity, fewer variables, and easier interpretability than the ML models [37].

While statistical analysis and ML share similarities, their predictive abilities may vary according to the characteristics of datasets [38]. Indeed, the benefit of ML in prognostic modeling may be dependent on factors like sample size, variable type, and even the disease investigated [36].

In this study, as in others [36,39], features were in a number exceedingly high over the number of patients to fully exploit the potential of ML and to give it a meaningful benefit over LR. In addition, it might be that a non-linear relationship exists between baseline and outcomes (cancer markers), which is not surprising given the great variability (e.g., genetic background, concomitant therapies) among all the patients included in the study independently of their specific OC condition.

Regarding the identification of the ionic species of interest, SELDI-TOF-MS does not provide the biochemical identity of the *m*/*z* peaks. However, while predisposing ad hoc experimental strategies for the biochemical identification of selected candidates, bioinformatics tools may provide in silico clues. As a proof of concept, we used ExPASy TagIdent [40] because it has already been used successfully for the in silico identity prediction of *m*/*z* peaks in similar studies of BM screening and the generation of diagnostic models from serum [41], tissue [42], and saliva [43]. Here, the analysis was performed by setting for each peak of interest an *m*/*z* interval of 0.1%, isoelectric point from 4 to 12, and Homo sapiens as the species of interest. The algorithm generated a list from which we selected proteins that are secreted, although proteins from other origins may well be introduced into the circulation. In this way, peak 29 (*m*/*z* 5.385) was associated with RAD51 isoform 2, which is involved in DNA damage repair and known to interact with BRCA2, which is a protein associated with familial predisposition to BC and OC [44]. Peak 41 (*m*/*z* 8.581) was associated with both the Serum amyloid A (SAA) 1 protein or the truncated form of Apolipoprotein (APO) A2. Both would be interesting to verify. SAA protein synthesis increases in response to tissue damage, infection, or inflammation and, based on proteomic studies, in several neoplasms (nasopharynx, kidney, stomach, liver, breast, endometrial tumors, melanoma) [45]. Lipoprotein metabolism is dysregulated in OC: APOA2, together with APOE, are independent classifiers of malignant OC [46]. Peak 56 (*m*/*z* 12.345) was associated with macrophage migration inhibitory factor (MIF), which is a pro-inflammatory cytokine involved in many chronic inflammatory and autoimmune diseases [47,48], which promotes tumor growth, metastasis and neo-angiogenesis [49]. MIF is overexpressed in breast cancer [50].

## 4. Materials and Methods

### 4.1. Recruitment and Participation of Human Subjects

The study was conducted in accordance with the Declaration of Helsinki and approved by the local Ethics Committee (Protocol no. 11168, of 11/07/2017, and amendment of 12/09/2018). All participants signed the informed consent.

Patients with documented diagnosis of OC and BC were enrolled at the Dipartimento Oncologico—Azienda USL Toscana Centro, Ospedale Santa Maria Annunziata, Bagno a Ripoli, Florence, Italy. Women with BC were recruited for the exclusion of non-specific gynecological tumor biomarkers.

Inclusion criteria were as follows:

- Women diagnosed with epithelial OC potentially undergoing radical surgery and who had not received previous chemotherapeutic or anti-hormonal treatments in the last four weeks;
- Women diagnosed with BC subjected to radical surgery and who had not yet started systemic treatments for the pathology.

Table 9 summarizes the available data regarding the subjects enrolled in the study. Full details for enrolled subjects are shown in Supplementary Table S1.

HS were enrolled through a screening campaign at the Institute for Cancer Research, Prevention and Clinical Network (ISPRO, Florence, Italy). HS did not have cancer of any kind and had not presented any oncological disease in the last five years, with the exception of in situ carcinoma of the cervix and skin, and did not have any significant systemic disease.

### 4.2. Preparation of Training and Testing Sets

A total of 147 individuals were selected (Supplementary Table S1): 50 OC patients; 49 BC patients; and 48 HS. Subjects were distributed among training and testing sets for statistical purposes (Table 9). Specifically, 35 OC, 34 BC, and 33 HS were assigned to the training set; the remaining 15 patients per group formed the testing set. Individuals in the training and testing groups were matched based on their age.

### 4.3. Collection of Saliva Samples

Unstimulated whole saliva samples were collected with sterile Falcon tubes (Merck Life Sciences, Milan, Italy), two tubes per woman, 3–5 mL of saliva per tube, between 9:00 and 11:00 am. Women were asked to refrain from eating, smoking and performing oral hygiene in the two hours before collection. After collection, samples were immediately frozen at −80 °C and shipped in dry ice to the laboratory in Pisa. Samples were then centrifuged at 3800 rcf (15 min, 4 °C) to remove mucus and cellular debris, aliquoted and stored at −80 °C again until analysis.

All samples were anonymized before further processing and a specific password-protected database was established to store clinical data.

### 4.4. Surface-Enhanced Laser Desorption Ionization-Time of Flight-Mass Spectrometry (SELDI-TOF-MS) Protein Profiling

Saliva samples were analyzed with SELDI-TOF-MS using ProteinChip Arrays (BioRad Laboratories, Segrate (MI), Italy). The surface chemistry of hydrophobic (H50), weak cation-exchange (CM10), strong anion-exchange (Q10) and immobilized metal affinity capture activated with copper (IMAC30-Cu^2+^) was tested to determine which one would yield the most informative ionic profile. All samples were loaded in duplicate. The initial screening revealed that the CM10 ProteinChip was the most informative, and therefore, it was selected for this study.

Protein chips were prepared as in [51,52]. All chemicals and plasticware below, unless specified differently, were obtained from Merck Life Sciences, Milan, Italy. Briefly, whole protein extracts were added to the required binding buffer (100 mM Na-Acetate, pH 4.0) and loaded onto pre-equilibrated spot surfaces. After incubation with horizontal shaking (60 min at room temperature, RT), the unbound proteins were washed thrice with the same binding buffer, salts were removed with HPLC-grade water; saturated Sinapinic acid (1 μL in 50% Acetonitrile/0.5% Trifluoroacetic acid) was added twice to each spot and allowed to dry. The reproducibility of SELDI-TOF-MS spectra from array to array on a single chip (intra-assay) and between chips (inter-assay) was checked by comparing the pooled saliva quality control sample at each run. Briefly, 500 μL was taken from five OC samples and five BC samples and mixed. The resulting 5 mL pool was divided into aliquots that were stored at −80 °C until use for SELDI-TOF-MS analysis. Ten cluster peaks, uniformly distributed for mass range and peak intensity, including statistically significant peaks, were used to calculate the coefficient of variance, both within and between assays, that is, 15% and 24% for intra- and inter-assay, respectively.

### 4.5. Data Acquisition and Analysis

Protein chips were analyzed with a linear TOF mass spectrometer (PCS 4000, BioRad Laboratories, Segrate (MI), Italy) using the following protocol: laser power 3500 nJ, matrix attenuation 1000, focus mass 10,000, sample rate 800 and 25% spot surface fired for ion profiling over a *m*/*z* range of 2000–50,000. Proteomics datasets were analyzed with ProteinChip Data Manager 3.5 software (provided with the hardware) as previously reported [51,52]. Variation of peak intensity (in microA) was assessed with a non-parametric Mann–Whitney U-test for two-group comparison.

### 4.6. Logistic Regression (LR)

The LR method was used for prediction model building, using IBM SPSS Statistics software, version 23 (IBM Italia, Segrate (MI), Italy). Validated salivary biomarkers were fit into LR models for each group comparison, and stepwise backward model selection was performed to determine final combinations of biomarkers. For each of these models, the predicted probability for each subject was obtained and used to construct the ROC curve to estimate the AUC and its 95% confidence interval. The sensitivity and specificity for the biomarker combinations were estimated by identifying the cut-off point of the predicted probability using the Youden index.

Validation was performed by applying the final combinations of biomarkers to independent samples and calculating sensitivity and specificity using the cut-off points of predicted probability identified in the model building procedure.

### 4.7. Variable Selection (VS) for Machine Learning (ML) Analysis

ML is a subset of artificial intelligence. Its methods involve bottom–up approaches that highlight the complex interactions between all variables while simultaneously eliminating those with minimal contribution to outcome prediction [53].

In ML, software recognizes patterns and creates data clusters with common characteristics that can influence outcomes. VS is a fundamental stage in ML model development, as it allows selecting input variables that most significantly affect the concerned target. This is a necessary step when the number of input variables is high compared to the number of available samples, which is frequent in many real-world applications, including those in medical research.

VS techniques can be divided in three main groups: filters, wrappers, and embedded approaches [54]. (1) The filter approach is a pre-processing step independent of the developed classifier. The variables subset was created by considering the association between input and output. Its main advantages are simplicity, speed and suitability to the treatment of large and complex databases. FUFES is an effective filter approach [55] which selects the most important input variables using a fuzzy logic-based approach [56]. (2) The wrapper approach estimates the performance of the model in order to select a subset of variables based on their predictive power. It considers the developed classifier as a black box without explaining how the algorithm works. Compared to the filter, it is more expensive from the computational point of view but more effective in terms of accuracy, as it is based on the performance of the selected model. In recent years, hybrid VS approaches have been introduced [57,58,59]. An example of the hybrid approach is proposed in [60], where the set of available variables is firstly reduced through a filter method, and then an exhaustive search is implemented in order to achieve a sub-optimal set of variables in a reasonable time. (3) The embedded approach integrates feature selection into the classifier algorithm. During the training phase, the classifier regulates its internal parameters and defines the suitable weights given for each feature to determine the best classification accuracy. A typical embedded approach is provided by DT [61,62,63].

The objective of the method proposed here was to automatically select the most suitable VS algorithm and, as a consequence, the best performing binary classifier based on the available data. The 77 variables obtained with SELDI-TOF-MS were used as input. Of note, with this approach, we did not apply any a priori knowledge about the specific nature of the data under analysis. The proposed logical procedure is schematized in Figure 4.

Three different binary subsets were considered: Subset (1) HS vs. OC + BC; Subset (2) OC vs. HS; Subset (3) HS vs. BC. Subset 1 was used to identify putative markers shared by the oncological patients OC and BC, whose specificity to one or the other cohort was to be highlighted by Subsets 2 and 3.

The dataset was pre-processed to eliminate outliers that can negatively affect the performance of the training procedure [63,64]. Then, the dataset was split into two groups: 75% of samples were used for training and the remaining 25% were used for testing. Finally, four different VS approaches were applied, and for each of them, the informative variables were selected. To improve stability, each VS algorithm was executed repeatedly by randomly varying every time the composition of the training dataset and including only the variables that were more frequently selected.

We adopted VS algorithms belonging to four different categories: filter, wrapper, embedded and hybrid. In particular, we applied the following methods: FUFES (filter); GIVE A GAP (wrapper); Decision Tree (DT, embedded). A hybrid method (filter + wrapper) was also used.

Four different classifiers were applied to each subset of selected variables: Bayesian classifier [65], Support Vector Machine (SVM) [66], Discriminant Analysis (DA)-based classifier [67] and DT [68].

For each subset and classifier, 100 iterations were performed by randomly varying the set of samples for training and testing at each iteration to overcome the eventual instability resulting from the VS. An average value of the classifier accuracy was computed.

The classifiers’ accuracy was measured in terms of BCR, which is a performance index widely adopted in the literature on binary classifiers because it can also be used with imbalanced datasets [69]. BCR is defined as the average value of two further indexes, namely sensitivity and specificity, which measure the proportion of correctly identified positive and negative samples.

BCR is computed as follows [70]:

$$BCR=\frac{1}{2}\left(sensitivity+specificity\right)=\frac{1}{2}\left(\frac{TP}{TP+FN}+\frac{TN}{TN+FP}\right)$$

where true positive (TP) and true negative (TN) represent the number of positive and negative samples correctly classified, while false negative (FN) and false positive (FP) represent the number of misclassified negative and positive samples, respectively.

## 5. Conclusions

In a meta-analysis by Ferraro et al., the specificity of CA125 for detecting OC was reported to be 78% [71]. Furthermore, according to Dochez et al., to date, the most efficient biological diagnostic tool to diagnose OC is in fact the combination of CA125 and HE4, with an AUC of 0.96, based on their review of markers for diagnosing OC, specifically HE4, CA 125, RMI and ROMA algorithms [72]. Taking into account the epidemiological characteristics of the disease, Charkhchi et al. claimed that due to the low prevalence of OC, the ideal screening test must have a sensitivity above 75% and a specificity of at least 99.6% [73]. Based on these findings, we propose that the signature of candidate BMs presented here, with sensitivity of 60% and specificity of 100% (Figure 3), shows interesting and promising potential not far from the ideal threshold, especially for its specificity. Of course, further studies and validation in the general population are necessary.

The comparison of the proteomic profile of saliva from HS with OC and BC patients, analyzed with LR and ML models, provided different sets of candidate BMs. This is both encouraging and intriguing; at any rate, the results in terms of both BCR and AUC show that the performances of LR and ML methods are similar even if the two approaches are completely different. Since the number of samples under study was small, ML methods could not be fully exploited. However, the fact that they are comparable with other less sophisticated methods leads us to think that although the size of the dataset is not ideal for these types of systems, they are still robust. We expect that the results, albeit already quite satisfactory, can drastically improve by increasing the number of patients. Furthermore, it is important to emphasize that the developed software is modular, does not require any a priori information and can already be tested as soon as data from other patients become available to improve accuracy.

On a biological level, further work is necessary to identify the biochemical nature of selected *m*/*z* peaks, and a whole validation phase will test their actual usefulness as OC BMs and possible application in stratifying patients. Yet ours and others’ work [23] make it possible to think of developing tools for the detection of the most appropriate BMs for screening purposes in the relevant population, using saliva as a safe, applicable, cheap body fluid that can be potentially gathered without medical intervention. This would allow the health care system to reach appropriate female individuals, even in remote areas, to collect samples for subsequent analysis in centralized, high-technology health hubs to improve OC diagnosis and save lives.

## Figures and Tables

**Table 1:** Input variables (*m*/*z* peaks) included in the study are designated by cardinal numbers for sake of simplification.

Peak No.	*m*/*z*	N°	*m*/*z*	N°	*m*/*z*
**1**	2.117	**26**	5.269	**52**	11.514
**2**	2.237	**27**	5.292	**53**	11.602
**3**	2.377	**28**	5.368	**54**	11.767
**4**	2.509	**29**	5.385	**55**	12.193
**5**	2.625	**30**	5.431	**56**	12.345
**6**	2.654	**31**	5.801	**57**	12.713
**7**	2.788	**32**	6.355	**58**	13.211
**8**	3.018	**33**	6.675	**59**	13.319
**9**	3.163	**34**	6.739	**60**	13.485
**10**	3.297	**35**	6.920	**61**	13.865
**11**	3.376	**36**	7.143	**62**	14.342
**12**	3.449	**37**	7.167	**63**	14.725
**13**	3.492	**38**	7.892	**64**	15.164
**14**	3.671	**39**	8.002	**65**	15.927
**15**	3.720	**40**	8.290	**66**	17.555
**16**	4.041	**41**	8.581	**67**	20.966
**17**	4.127	**42**	9.990	**68**	21.692
**18**	4.139	**43**	10.116	**69**	22.395
**19**	4.370	**44**	10.213	**70**	23.578
**20**	4.426	**45**	10.304	**71**	24.346
**21**	4.547	**46**	10.467	**72**	25.333
**22**	4.577	**47**	10.683	**73**	25.709
**23**	4.929	**48**	10.864	**74**	26.056
**24**	5.226	**49**	11.038	**75**	27.855
**25**	5.244	**50**	11.253	**76**	28.169
		**51**	11.382	**77**	28.816

**Table 2:** Peaks differentially expressed in the training set. The sign of differential expression in cohorts of samples compared one to one is represented. Stars: statistical significance according to non-parametric Mann–Whitney U test. ****: *p* = 0; ***: *p* < 0.001; **: *p* < 0.01; *: *p* < 0.05; Ns: not significant. Cardinal numbers of peaks refer to *m*/*z* species listed in Table 1.

Peak No.	OC vs. HS	OC vs. BC	BC vs. HS
2	Ns	* OC > BC	Ns
9	** OC > HS	** OC > BC	Ns
13	* OC < HS	Ns	Ns
16	Ns	Ns	* BC < HS
20	** OC < HS	Ns	**** BC < HS
22	* OC < HS	Ns (OC ≥ BC)	** BC < HS
27	Ns	Ns	* BC < HS
28	* OC > HS	** OC > BC	Ns
29	* OC > HS	* OC > BC	Ns
32	** OC < HS	Ns	* BC < HS
34	*** OC < HS	Ns	** BC < HS
38	Ns	* OC > BC	Ns
41	** OC > HS	Ns	Ns
43	Ns	Ns	* BC < HS
44	** OC < HS	Ns	** BC < HS
45	* OC < HS	Ns	* BC < HS
46	** OC < HS	Ns	** BC < HS
47	* OC < HS	Ns	* BC < HS
48	** OC < HS	* OC < BC	Ns
56	* OC < HS	Ns	** BC < HS
57	*** OC < HS	** OC < BC	Ns
58	** OC < HS	Ns	** BC < HS
59	* OC < HS	Ns	** BC < HS
60	** OC < HS	Ns	** BC < HS
63	Ns	Ns	* BC < HS
64	* OC < HS	Ns	Ns
67	*** OC < HS	Ns	** BC < HS
68	** OC < HS	Ns	* BC < HS
70	Ns	Ns	* BC < HS
71	** OC < HS	Ns	** BC < HS
72	*** OC < HS	Ns	** BC < HS
73	Ns	Ns	* BC < HS
74	* OC < HS	Ns	** BC < HS

**Table 3:** Peaks differentially expressed in the testing set. The sign of differential expression in cohorts of samples compared one to one is represented. Stars: statistical significance according to non-parametric Mann–Whitney U tests. ****: *p* = 0; ***: *p* < 0.001; **: *p* < 0.01; *: *p* < 0.05; Ns: not significant. Cardinal numbers of peaks refer to *m*/*z* species listed in Table 1.

Peak No.	OC vs. HS	OC vs. BC	BC vs. HS
17	Ns	Ns	* BC < HS
20	Ns	Ns	* BC < HS
22	Ns (OC < HS)	Ns	Ns
25	Ns	Ns	* BC > HS
30	Ns	Ns	* BC > HS
33	** OC < HS	Ns	** BC < HS
34	*** OC < HS	Ns	* BC < HS
38	Ns	Ns	* BC < HS
48	Ns	* OC < BC	Ns
49	Ns	* OC < BC	Ns
54	Ns	* OC < BC	** BC > HS
56	Ns (OC < HS)	Ns	* BC < HS
58	* OC < HS	Ns	**** BC < HS
59	* OC < HS	Ns	**** BC < HS
60	* OC < HS	Ns	* BC < HS
63	* OC > HS	Ns	Ns
65	Ns	** OC < BC	Ns
66	Ns	** OC < BC	Ns

**Table 4:** Classification performance concerning Subset 1 (HS vs. OC + BC).

Classifier	Index	VS Approach			
		Filter	Wrapper	Embedded	Hybrid (Filter/Wrapper)
Bayes	BCR/devStd Sensitivity Specificity AUC	0.64/0.06 0.85 0.42 0.65	0.54/0.08 0.36 0.72 0.51	0.57/0.08 0.42 0.68 0.72	0.54/0.04 0.95 0.12 0.55
SVM	BCR/devStd Sensitivity Specificity AUC	0.59/0.05 0.95 0.22 0.69	0.60/0.07 0.95 0.26 0.69	0.55/0.06 0.89 0.21 0.77	0.59/0.03 0.98 0.04 0.62
DA	BCR/devStd Sensitivity Specificity AUC	0.61/0.07 0.91 0.31 0.68	0.51/0.03 0.96 0.06 0.63	0.58/0.07 0.89 0.26 0.78	0.60/0.06 0.93 0.27 0.68
DT	BCR/devStd Sensitivity Specificity AUC	0.62/0.08 0.78 0.45 0.67	0.65/0.06 0.79 0.51 0.62	0.74/0.08 0.85 0.63 0.72	0.59/0.08 0.78 0.39 0.68

**Table 5:** Classification performance concerning Subset 2 (OC vs. HS).

Classifier	Index	VS Approach			
		Filter	Wrapper	Embedded	Hybrid (Filter/Wrapper)
Bayes	BCR/devStd Sensitivity Specificity AUC	0.67/0.08 0.84 0.49 0.70	0.51/0.09 0.55 0.47 0.54	0.65/0.09 0.61 0.69 0.76	0.62/0.10 0.83 0.42 0.61
SVM	BCR/devStd Sensitivity Specificity AUC	0.71/0.09 0.82 0.60 0.75	0.59/0.07 0.57 0.60 0.52	0.69/0.08 0.75 0.63 0.81	0.63/0.08 0.80 0.46 0.72
DA	BCR/devStd Sensitivity Specificity AUC	0.68/0.08 0.80 0.56 0.76	0.66/0.08 0.56 0.75 0.54	0.69/0.08 0.76 0.62 0.86	0.64/0.09 0.80 0.47 0.77
DT	BCR/devStd Sensitivity Specificity AUC	0.65/0.10 0.65 0.65 0.70	0.62/0.10 0.62 0.62 0.65	0.73/0.07 0.73 0.73 0.77	0.64/0.08 0.61 0.67 0.65

**Table 6:** Classification performance concerning Subset 3 (BC vs. HS).

Classifier	Index	VS approach			
		Filter	Wrapper	Embedded	Hybrid (Filter/Wrapper)
Bayes	BCR/devStd Sensitivity Specificity AUC	0.65/0.07 0.46 0.83 0.78	0.61/0.08 0.44 0.78 0.50	0.66/0.08 0.49 0.83 0.66	0.59/0.07 0.24 0.94 0.75
SVM	BCR/devStd Sensitivity Specificity AUC	0.66/0.08 0.59 0.74 0.82	0.53/0.08 0.52 0.54 0.58	0.76/0.07 0.71 0.80 0.70	0.65/0.08 0.55 0.76 0.77
DA	BCR/devStd Sensitivity Specificity AUC	0.66/0.09 0.56 0.77 0.80	0.50/0.09 0.46 0.53 0.71	0.70/0.09 0.60 0.79 0.68	0.63/0.09 0.52 0.74 0.77
DT	BCR/devStd Sensitivity Specificity AUC	0.70/0.10 0.67 0.72 0.69	0.78/0.09 0.75 0.81 0.62	0.79/0.07 0.79 0.78 0.70	0.65/0.09 0.63 0.67 0.65

**Table 7:** Classification performance on whole dataset.

Classifier		HS/OC + BC	HS/OC	HS/BC
Bayes	BCR DevSt Sens. Spec. AUC	0.61 0.07 0.56 0.66 0.65	0.56 0.08 0.51 0.61 0.63	0.60 0.08 0.50 0.67 0.57
SVM	BCR DevSt Sens. Spec. AUC	0.63 0.07 0.76 0.50 0.70	0.61 0.09 0.58 0.63 0.66	0.72 0.07 0.69 0.75 0.76
DA	BCR DevSt Sens. Spec. AUC	0.56 0.08 0.73 0.40 0.62	0.50 0.10 0.51 0.49 0.51	0.55 0.11 0.57 0.54 0.55
DT	BCR DevSt Sens. Spec. AUC	0.64 0.08 0.80 0.49 0.68	0.64 0.1 0.64 0.64 0.69	0.71 0.10 0.74 0.67 0.72

**Table 8:** Summary of results and the percentage gain in terms of performance obtained using the VS algorithm.

Dataset	Selected Variables	BCR (Embedded–DT)	BCR (All Variables)	% Gain
OC + BC/HS	2-3-12-14-16-17-18-19-20-21-22-34-41-46-49-54-68-72	0.74	0.64	13.5%
OC/HS	3-7-20-34-40	0.73	0.64	12.3%
BC/HS	19-20-21-22-30-34-49-54-59	0.79	0.71	0.10%

**Table 9:** Summary of demographic and clinical pathological characteristics of the participants in the study subdivided into training and testing sets. All women were of Caucasian ethnicity.

	Total Numerosity	Numerosity of the Training Set	Numerosity of the Testing Set
Healthy Women (HS)	48	33	15
Age (range)	45–73	45–73	49–73
Age (mean)	61.67	60.54	62.80
Ovarian Cancer (OC) Patients	50	35	15
Age (range)	43–84	43–84	49–80
Age (mean)	62.49	62.18	62.80
Serous		23	7
Other (see Supplementary Table S1)		12	7
Metastasis at diagnosis		12	8
Breast Cancer (BC) Patients	49	34	15
Age (range)	34–87	34–87	46–77
Age (mean)	61.26	59.71	62.80
Ductal		22	9
Lobular		6	3
Other (see Supplementary Table S1)		5	3
Metastasis at diagnosis		14	4

## Data Availability

The data presented in this study are available on request from the corresponding author.

## Supplementary Materials

The supporting information can be downloaded at: https://www.mdpi.com/article/10.3390/ijms242115716/s1.
