# Supplementary material for: Top–Down Proteomics of Human Saliva, Analyzed with Logistic Regression and Machine Learning Methods, Reveal Molecular Signatures of Ovarian Cancer
Source: Int J Mol Sci. 2023 Oct 28;24(21):15716. doi: 10.3390/ijms242115716 (PMC10648137; doi:10.3390/ijms242115716)
Supplement: Supplementary file 1 [file ijms-24-15716-s001.zip › ijms-2613557-supplementary.pdf]

Supplementary Table S1. Age and clinical pathological characteristics of the participants to the study, subdivided into Training and Testing sets.

DCIS: ductal carcinoma in situ. LCIS: lobular carcinoma in situ. NE: neuroendocrine. MaC: metastasis at time of sample collection. TNM: Classification of Malignant Tumours, by the Union for International Cancer Control. Empty cells denote absence of data.

Patient No.	Patient ID code	AGE	MaC	DIAGNOSIS	GRADE	T	N	M	STAGE
**TRAINING SET**				**OVARIAN CANCER**					
1	4OC	77		serous	3				
2	7OC	67	x	serous	3	3c	0	X	IIIC
3	9OC	48	x	serous	3	3	1	X	IIIC
4	10OC	43	x	serous	3	3	x	X	IIIB
5	22OC	45	x	solid	3	3	1	X	IIIC
6	23OC	66	x	serous	3	3c	1	X	IIIA
7	24OC	69	x	serous	3	1	1	X	IIIA
8	25OC	56	x	serous	3	3c	1		IIIA
9	26OC	64		solid-tubular, endometrioid	3				
10	27OC	59	x	serous					
11	30OC	77	x	serous					
12	34OC	84	x	solid					
13	35OC	52		serous					
14	36OC	49	x	solid	3	3	1		IIIA
15	49OC	47		serous	3				IIIC

16	50OC	61	x	serous	3				
17	53OC	55	x	serous	3				
18	59OC	71		serous					IIIC
19	63OC	76		serous	3				
20	67OC	68		endometrioid	2	1c	0	X	IA
21	71OC	60	x	serous	1				
22	81OC	66		serous	1				
23	84OC	71		clear cells		1c			
24	89OC	46	x	serous	3	3c	1		IIIC
25	104OC	70		undifferentiated					
26	106OC	43		serous	3	3	X	X	IIIB
27	107OC	55		serous	2	3b	X	X	IIIB
28	113OC	73		clear cells					
29	116OC	61		serous	3	3b	0	X	
30	136OC	75		high grade carcinoma	3	3b	X	X	III
31	137OC	78		serous	3	2b	0	X	IIB
32	138OC	62		high grade carcinoma		3c			IIIC
33	141OC	71		serous					
34	142OC	69		carcinoma					
35	144OC	57		high grade carcinoma		1b	X		

TESTING SET

1	117OC	53		granulosa	3				
2	121OC	51		endometrioid	3				
3	122OC	60		serous	3	2a	1	0	IIIC
4	125OC	71		serous					
5	126OC	71		serous	3	3c	0	X	
6	129OC	69		carcinoma					
7	134OC	53		carcinosarcoma	3				
8	235OC	65	x	serous	3	3b		X	IV
9	240OC	50		carcinoma	3	2b	0		IIB
10	243OC	67		serous		1c2	X		IC
11	245OC	54		carcinoma	3	3c	X		
12	247OC	65		serous	3	3b	0		IIIB
13	248OC	81		serous	3	3c			IIIC
14	252OC	70		serous	3				
15	253OC	67		carcinoma	3	3b			IIIB

TRAINING SET **BREAST CANCER**

1	1BC	56		Ductal	3	1c	0		IA
2	2BC	69		Ductal	3	2	0		IIA

3	3BC	51	x	Ductal	3	2mic	3a	X	IIIC
4	5BC	79	x	Lobular	1	3	1a		IIIA
5	6BC	67		Ductal	2	1bmic	0		IA
6	8BC	39	x	Ductal	2	2 mic	X	X	
7	11BC	78	x	Ductal	2	1c	1	0	IIA
8	12BC	57		Ductal	3	1c	0	X	IA
9	13BC	87		infiltrating, with NE features	2	2	1a		IIB
10	14BC	50		Ductal	2	2	1a		IIB
11	15BC	77		Lobular	2	1c	0		IA
12	186BC	51		Ductal	2	2	3	X	IIIC
13	17BC	39		Ductal+DCIS	2	1c mic	1 mic		IB
14	18BC	82		Colloidal+DCIS	2	1b mic	0		IA
15	19BC	64		Lobular+DCIS+LCIS	2	1c	0	X	IA
16	20BC	54		Ductal+DCIS	3	1c	1mic		IB
17	21BC	55		Ductal+DCIS	3	2	3	X	IIIC
18	28BC	34		Ductal+DCIS	2	1c mic	1 mic	X	IB
19	29BC	52		Ductal	1	2	1a	X	IIB
20	31BC	68		Ductal	3	1c	0	X	IA
21	32BC	65		Lobular	2	2mic	2	X	IIIA
22	22BC	56	x	Ductal+lobular	3	2mic	2a	X	IIIA

23	37BC	67							
24	38BC	69		Ductal	1	1c	0		IA
25	39BC	62		Lobular	2	2 mic	3a		IIIC
26	40BC	65		Lobular	2	1c	0		IA
27	41BC	70		Ductal+DCIS	3	1c	0		IA
28	42BC	52		Ductal+lobular+DCIS	2	1a mic	1mic	X	IB
29	43BC	50		Ductal+DCIS	3	1c	1mic	X	IB
30	44BC	71		Ductal+DCIS	2	1b	0		IA
31	93BC	55		Lobular	3	1c mic	0	X	IA
32	94BC	57		Ductal	3	1c	0		IA
33	95BC	41		Ductal					
34	97BC	67		Ductal+DCIS	2	1a	1	0	IIA
**TESTING SET**									
1	45BC	57		Ductal	1	1a mic	0		IA
2	46BC	70		Lobular	2	1c	1	X	IIA
3	47BC	71		Ductal	2	2	1a		IIB
4	51BC	61		Ductal+DCIS	2	1 mic	3		IIIC
5	52BC	52		Lobular+LCIS	2	2	1a		IIB
6	56BC	69	x	Ductal+lobular	2	1b			
7	58BC	59		Ductal+DCIS	2	2	1	X	IIB

8	60BC	77	x	infiltrating					
9	61BC	67		Left: ductal; right: lobular+LCIS	Right:2; left: 2	Right: 1c; left: 2	Right: 0; left: 1mic		IA dx IIB sx
10	62BC	74		Ductal+DCIS	1	1b	0		IA
11	68BC	46		Ductal	2	1b mic	1a	X	IIA
12	69BC	54		Ductal	3	2	0	X	IIA
13	72BC	64		Lobular	1	1b	0		IA
15	73BC	53		Ductal+DCIS	3	1c	0		IA
15	80BC	68		Ductal	3	IS			
